# First Reported Case of Acute Kidney Injury Following Intraureteral Indocyanine Green Administration During Bilateral Endometrioma Excision

**DOI:** 10.3390/jcm14248758

**Published:** 2025-12-10

**Authors:** Anna Scholz, Olga Redko, Michał Kostrzanowski, Filip Dąbrowski

**Affiliations:** 1 Department of Obstetrics, Perinatology and Neonatology, Center of Postgraduate Medical Education, 01-809 Warsaw, Poland; 2 Department of Gynecology, Bielański Hospital, 01-809 Warsaw, Poland; mail2lira@gmail.com (O.R.); mkostrzan@gmail.com (M.K.); 3 Department of Gynecological Oncology, Center of Postgraduate Medical Education, 01-809 Warsaw, Poland; fil.dabrowski@gmail.com

**Keywords:** indocyanine green, acute kidney injury, endometrioma, ureter visualisation, fluorescence-guided surgery, case report

## Abstract

Indocyanine green (ICG) is widely used in minimally invasive surgery for real-time fluorescence imaging of vascular, biliary, and urological structures. Although its intravenous use has been extensively validated, data on intraureteral administration remain scarce, particularly regarding renal safety. We report the case of a 50-year-old woman undergoing laparoscopic bilateral endometrioma excision with intraureteral ICG instillation for ureteral visualisation. Despite an uneventful surgery, the patient developed anuria and acute kidney injury (AKI) within 24 h, requiring temporary hemodialysis. Imaging demonstrated bilateral renal dysfunction without evidence of ureteral transection. Renal function gradually improved with supportive care, and dialysis was discontinued. This is, to our knowledge, the first reported case of AKI following intraureteral ICG use. Potential mechanisms include dye-induced tubular toxicity, ischemic injury, and multifactorial perioperative stressors. Given the increasing adoption of near-infrared fluorescence in gynecologic and urologic surgery, our case highlights the urgent need for systematic studies on the renal safety of intraureteral ICG administration. Until further evidence emerges, surgeons should use the technique with caution, particularly in patients with preexisting risk factors for AKI.

## 1. Introduction

Indocyanine green (ICG) is a near-infrared fluorescent dye widely used in minimally invasive surgery for vascular and biliary mapping. Its intravenous safety profile is well established, yet data on intraureteral administration remain scarce, especially concerning renal safety. We present the first case of acute kidney injury (AKI) following intraureteral ICG use during laparoscopic excision of ovarian endometriomas, highlighting a potential nephrotoxic mechanism

Endometriosis is a chronic estrogen-dependent disease affecting about 10% of women of reproductive age [1]. When ovarian involvement causes pain, infertility, or suspicion of malignancy, surgical excision is often required [2]. Laparoscopic techniques offer faster recovery and better visualization [3], yet carry a 0.3–1.5% risk of ureteral injury, especially in cases with dense adhesions or distorted pelvic anatomy [4,5]. Traditional strategies to reduce ureteral injury include preoperative stent placement, intraoperative cystoscopy with ureteral jet visualisation, or the use of lighted ureteral catheters [6]. While effective, these approaches may be invasive, costly, or technically challenging. Recently, fluorescence-guided surgery using indocyanine green (ICG) has emerged as a promising alternative.

ICG is a tricarbocyanine dye that binds plasma proteins and fluoresces in the near-infrared (NIR) spectrum when excited by appropriate light sources [7]. Since its introduction in 1954, it has been widely used for measuring cardiac output, assessing hepatic function, and performing retinal angiography [8]. In surgical oncology, ICG fluorescence has enabled the mapping of sentinel lymph nodes, the identification of the biliary tract, and the assessment of perfusion [9,10,11]. Its safety profile after intravenous injection is well established, with an incidence of severe adverse reactions <0.05% [12].

In urology, ICG fluorescence has been increasingly applied for ureteral visualisation during complex pelvic surgery [13,14,15]. Because systemic ICG does not directly perfuse the ureters, intraureteral instillation has been proposed to achieve real-time NIR visualisation. Early reports demonstrated feasibility and safety in colorectal resections and hysterectomies [16,17,18]. However, pharmacokinetic data on intraureteral administration are sparse, and renal safety outcomes have not been systematically studied.

To date, no published cases have described acute kidney injury (AKI) directly associated with intraureteral ICG administration. AKI is a serious perioperative complication related to increased morbidity, prolonged hospitalisation, and higher long-term risk of chronic kidney disease [19,20]. Identifying potential iatrogenic causes is therefore critical.

Here, we present the first reported case of AKI requiring dialysis after intraureteral ICG instillation during laparoscopic bilateral endometrioma excision. We discuss possible pathophysiological mechanisms, review the relevant literature, and highlight implications for surgical practice and future research. To verify novelty, we performed a systematic search in PubMed, Scopus, and Web of Science (search date: July 2025) using the terms ‘indocyanine green’, ‘intraureteral’, ‘ureteral instillation’, ‘renal failure’, ‘acute kidney injury’, and ‘nephrotoxicity’. No reports of AKI temporally associated with intraureteral ICG were identified. Existing publications describe feasibility and safety without renal complications.

## 2. Case Presentation

### 2.1. Patient Background

A 50-year-old woman (gravida 0, para 0) with a history of symptomatic bilateral ovarian endometriomas was admitted for elective laparoscopic excision. She reported chronic pelvic pain and menorrhagia refractory to medical management. Her past medical history was notable for paranoid schizophrenia, for which she was not taking any medication at the time of admission. She had no history of renal disease, hypertension, diabetes, or allergies. Baseline laboratory evaluation revealed normal renal function (serum creatinine 0.84 mg/dL, estimated glomerular filtration rate [eGFR, CKD-EPI 2021] > 90 mL/min/1.73 m^2^), normal liver enzymes, and a haemoglobin level of 11.6 g/dL. Preoperative imaging (transvaginal ultrasound and MRI) demonstrated bilateral ovarian cysts consistent with endometriomas, measuring 6.5 cm on the left and 5.0 cm on the right. No hydronephrosis or urinary tract anomalies were noted. Intraureteral (retrograde) administration of indocyanine green (ICG) is not formally registered for this indication in Poland. However, the Center of Postgraduate Medical Education, as a university hospital, conducts several approved institutional projects evaluating intraoperative applications of ICG. For the present procedure, written informed consent was obtained from the patient both for participation in the ICG program and for publication of this case, including acknowledgement of potential renal risks.

### 2.2. Surgical Procedure

The patient underwent laparoscopy under general anaesthesia. Pneumoperitoneum was established with CO_2_ at 12 mmHg. Dense adhesions were noted between the ovaries, uterus, and pelvic sidewall. To minimise ureteral injury, the surgical team elected to use intraureteral ICG for real-time ureter visualisation. ICG (Diagnogreen^®^, Daiichi Sankyo Co., Tokyo, Japan) was diluted to 0.5 mg/mL; 5 mL was instilled per ureter (total 5 mg) after installing ureteral catheters. After fluorescence confirmation, ureteral catheters were removed immediately. A 16F Foley catheter was maintained intraoperatively, and urine output was 120 mL during the procedure. No iodinated contrast was used under fluoroscopy. The patient did not receive nephrotoxic agents such as NSAIDs or aminoglycosides perioperatively; analgesia consisted of paracetamol and low-dose opioids. Near-infrared fluorescence (NIRF) imaging (Karl Storz IMAGE1 S system) confirmed prompt ureteral visualisation, aiding in dissection. The procedure proceeded uneventfully with bilateral endometrioma excision and preservation of ovarian tissue. Estimated blood loss was 400 mL. Operative time was 120 min. The patient was hemodynamically stable throughout, with no episodes of hypotension; mean arterial pressure remained ≥70 mmHg. She exhibited a low baseline blood pressure (approximately 95/60 mmHg), consistent with her usual preoperative measurements.

### 2.3. Postoperative Course

In the first 12 h, urine output was <50 mL, progressing to anuria by postoperative day (POD) 1. Serum creatinine rose from baseline 0.84 mg/dL to 2.35 mg/dL, accompanied by anuria within 24 h, fulfilling KDIGO criteria for acute kidney injury. This was accompanied with elevated urea (68.9 mg/dL) and reduced eGFR (21.9 mL/min/1.73m^2^). Inflammatory markers were moderately increased (C-reactive protein [CRP] 35.5 mg/L). Preoperative serum electrolytes were within normal ranges (Na^+^ 139 mmol/L, K^+^ 4.3 mmol/L). On POD 1, potassium increased to 5.6 mmol/L and sodium decreased slightly to 135 mmol/L, consistent with AKI-related electrolyte imbalance.

A nephrology consultation was obtained. Despite fluid resuscitation and diuretic challenge, anuria persisted. On POD 2, creatinine further increased to 2.7 mg/dL with hyperkalemia (5.6 mmol/L), prompting initiation of hemodialysis.

### 2.4. Imaging and Diagnostic Workup

Renal ultrasound revealed normal kidney size and cortical thickness, without hydronephrosis or obstruction. A contrast-enhanced CT urogram (POD 2) showed bilateral delayed nephrograms with minimal excretion, but no ureteral transection or leak. Mild pelvic hematoma and postoperative oedema were noted. Findings were consistent with bilateral renal dysfunction rather than mechanical obstruction.

### 2.5. Recovery and Outcome

Dialysis was required on two consecutive days. From POD 5, urine output gradually returned, reaching 1200 mL/day by POD 6. Serum creatinine declined to 1.35 mg/dL by POD 7 and normalised (0.92 mg/dL) by POD 9.

The patient was discharged on POD 9 with normal renal function, stable haemoglobin, and complete recovery. The patient remains on follow-up, and the renal function is normal. She provided written informed consent for publication of this case.

### 2.6. Timeline Summary

Clinical course of the patient and laboratory trends were summarized in Table 1. 

## 3. Discussion

### 3.1. Indocyanine Green: Pharmacology and Applications

ICG is a water-soluble anionic dye that binds plasma proteins and is exclusively eliminated by the liver into bile without enterohepatic recirculation [7]. Its half-life is 3–5 min after intravenous injection. Because it does not undergo renal clearance, systemic toxicity is rare, and it has been considered safe even in renal impairment [12].

In surgery, NIRF with ICG enhances visualisation of vascular perfusion, biliary anatomy, and lymphatic drainage [9,10,11]. Urologic and gynecologic applications have multiplied, with intraureteral ICG offering a minimally invasive alternative to stents or catheters for ureter identification [13,14,15,16,17,18].

### 3.2. Literature on Intraureteral ICG Use

Several case series and feasibility studies have reported the successful administration of intraureteral ICG. Siddighi et al. first demonstrated its use during robotic hysterectomy, with excellent visualisation and no adverse events [16]. Barnes et al. confirmed the feasibility of this approach during colorectal resections [17]. More recently, Jansen et al. conducted a prospective series of 30 patients undergoing gynecologic laparoscopy with intraureteral ICG, reporting no renal complications [18]. However, these studies were limited by small sample size and short follow-up, and renal function was not systematically monitored.

To verify novelty, we performed a systematic search in PubMed, Scopus, and Web of Science (search date: July 2025) using the terms ‘indocyanine green’, ‘intraureteral’, ‘ureteral instillation’, ‘renal failure’, ‘acute kidney injury’, and ‘nephrotoxicity’. No reports of AKI temporally associated with intraureteral ICG were identified. Existing publications describe feasibility and safety without renal complications. To our knowledge, no previous reports have described AKI temporally associated with intraureteral ICG instillation.

### 3.3. Potential Mechanisms of AKI in This Case

The development of acute kidney injury (AKI) within 24 h of surgery, temporally associated with intraureteral indocyanine green (ICG) instillation, raises concern for a possible causal link. Although the patient ultimately recovered renal function, the episode of anuria was severe enough to necessitate dialysis, underscoring the clinical significance. Several potential mechanisms, either individually or in combination, may explain the renal insult in this case.

#### 3.3.1. Direct Tubular Toxicity from ICG

ICG is not cleared by the kidneys after intravenous administration, as it is exclusively taken up by hepatocytes and excreted into the bile [7,12]. However, intraureteral delivery bypasses hepatic metabolism and directly exposes the urothelium, renal pelvis, and potentially renal tubules to the dye. The extent of retrograde diffusion of ICG into renal parenchyma is not well studied.

Preclinical data indicate that cyanine dyes, including ICG, can generate reactive oxygen species and mitochondrial dysfunction at high concentrations [21]. In vitro studies have demonstrated dose-dependent cytotoxicity to renal tubular epithelial cells when exposed to structurally related dyes [21]. It is conceivable that the retrograde diffusion of ICG, particularly at the instilled concentration, may have caused direct tubular epithelial injury, manifesting as acute tubular necrosis.

**Figure 1:**
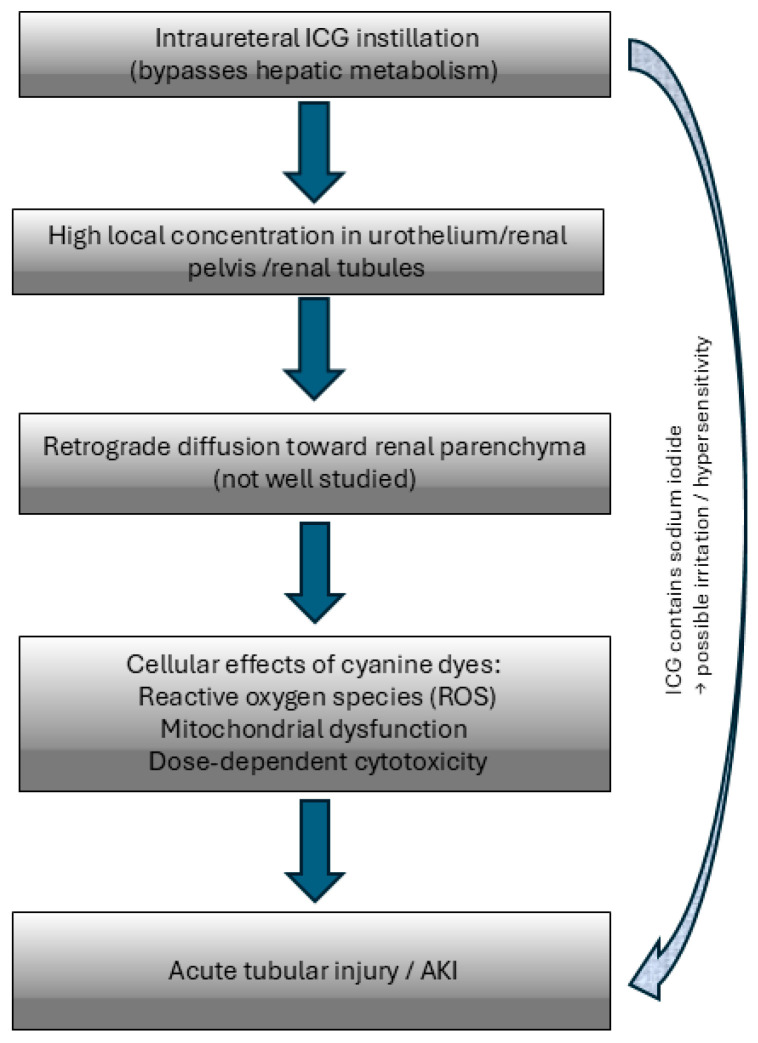
Proposed mechanism of acute kidney injury following intraureteral indocyanine green (ICG) instillation.

Furthermore, ICG contains sodium iodide as a stabiliser. Although rare, hypersensitivity or chemical irritation of renal tissue has been reported with iodine-containing compounds [7]. While systemic allergic manifestations were absent in our patient, a local toxic effect on the urothelium cannot be excluded. Figure 1.

#### 3.3.2. Ischemic Injury Related to Surgical Factors

AKI in laparoscopic surgery is frequently multifactorial, with hemodynamic and ischemic contributors. Pneumoperitoneum at intra-abdominal pressures ≥ 12 mmHg can reduce renal blood flow, increase renal vascular resistance, and impair glomerular filtration [22,23]. Prolonged surgical duration may amplify these effects.

Our patient underwent a 120 min laparoscopy with CO_2_ pneumoperitoneum at a pressure of 12 mmHg. Although intraoperative records documented stable mean arterial pressure, she had a baseline low blood pressure (95/60 mmHg), which may have reduced her renal autoregulatory reserve. Even modest reductions in perfusion pressure during surgery could have tipped the renal oxygen supply–demand balance toward ischemia.

Additionally, a perioperative drop in haemoglobin from 11.6 g/dL to 9.8 g/dL reflects blood loss-induced anaemia. Experimental studies show that anaemia exacerbates susceptibility of the kidney to hypoxic damage during surgical stress [22]. Together, pneumoperitoneum, low baseline blood pressure, and anaemia could have potentiated ischemic tubular injury.

#### 3.3.3. Contrast-Induced Nephropathy Versus Confounding

Contrast-induced nephropathy (CIN) is a well-recognised complication of iodinated contrast exposure [24]. Our patient underwent CT urography on postoperative day 1; however, anuria was established before imaging, making CIN an unlikely primary cause. Nonetheless, contrast exposure may have compounded existing injury and delayed recovery. This highlights the diagnostic dilemma when multiple nephrotoxic exposures occur perioperatively.

#### 3.3.4. Multifactorial Perioperative Stress Response

Modern concepts of AKI recognise the syndrome as the result of multiple “hits” rather than a single insult [19,20]. Surgical stress induces systemic inflammation, neurohormonal activation, and oxidative stress, all of which sensitise the kidney to injury [25]. In this patient, intraureteral ICG exposure may have acted as a novel nephrotoxic trigger, superimposed on background risk factors including low blood pressure, pneumoperitoneum, anaemia, and surgical inflammation.

This multifactorial model explains why most patients tolerate intraureteral ICG uneventfully, but a susceptible individual may experience AKI. It also highlights the importance of patient-specific factors, including preexisting renal reserve, comorbidities, and intraoperative hemodynamics.

#### 3.3.5. The Case for ICG-Related Causality

Several considerations suggest that intraureteral ICG may have contributed to the renal dysfunction observed in this case. The close temporal association is notable, as anuria developed within hours of dye instillation, before exposure to other recognised nephrotoxins. The bilateral nature of the injury, affecting both kidneys simultaneously, is also consistent with either a systemic factor or a bilateral local insult, rather than a unilateral mechanical obstruction. Importantly, imaging ruled out obstruction or ureteral transection as a cause, and the subsequent full recovery of renal function supports an acute, potentially reversible toxic or ischemic insult rather than permanent parenchymal damage. Taken together, these observations raise concern that intraureteral ICG exposure may have contributed to the development of acute kidney injury in this patient. However, a definitive causal relationship cannot be established.

### 3.4. Comparison with Other Ureter-Visualising Dyes

Alternative dyes include methylene blue and indigo carmine. Both have been associated with rare but documented nephrotoxicity and allergic reactions [26,27]. By contrast, ICG was considered safer due to hepatic clearance. However, intraureteral exposure may bypass hepatic metabolism, raising new concerns about safety.

### 3.5. Clinical Implications

Recent systematic analyses confirm that intravenous ICG is safe even in patients with advanced chronic kidney disease or kidney transplantation [28], and that adverse reactions, though uncommon, have been well characterized [29]. However, these data pertain exclusively to systemic exposure. In contrast, intraureteral administration bypasses hepatic metabolism and directly exposes the urothelium and renal pelvis to the dye, a pathway not evaluated in the existing safety literature.

This case underscores both the potential advantages and the hidden risks of intraureteral ICG instillation in complex gynecologic surgery. In the setting of advanced endometriosis or large adnexal masses, accurate ureteral identification is crucial to prevent iatrogenic injury. Ureteral damage remains one of the most feared complications of gynecologic laparoscopy, with reported incidences ranging from 0.5% to 2% in high-complexity procedures [6]. Fluorescence guidance offers an appealing adjunct, enhancing visual contrast and providing real-time intraoperative navigation [6,10]. However, as our experience illustrates, novel routes of ICG administration—particularly those that bypass traditional pharmacokinetic pathways—may carry unforeseen risks.

Published feasibility studies report intraureteral ICG concentrations ranging from 0.125 mg/mL to 2.5 mg/mL. Until pharmacokinetic data are available, doses ≤2.5 mg per ureter appear sufficient for adequate fluorescence [3]. However, no standardised regimen exists for intraureteral application. Published pilot studies have varied widely in their approach, instilling anywhere from 1 to 5 mL of ICG solution, often at concentrations extrapolated from intravenous protocols [10,11]. The lack of harmonisation not only complicates comparison across studies but also increases the risk of accidental overdosing. Our patient received a relatively small total volume (2 mL), yet still developed significant AKI. This raises the possibility that even small amounts may achieve locally nephrotoxic concentrations, particularly in individuals who are susceptible. Future investigations must therefore focus on establishing safe thresholds for urinary tract exposure, ideally supported by preclinical toxicological studies. Patients with chronic kidney disease, diabetes, or low baseline blood pressure should be considered at increased risk for AKI and monitored postoperatively with daily serum creatinine and urine output. We recommend pre- and postoperative electrolyte testing and early nephrology consultation if urine output remains <0.5 mL/kg/h for >6 h.

A second implication is patient selection. Not all individuals may tolerate intraureteral dye exposure equally. Our patient had a naturally low baseline blood pressure, modest anaemia, and prolonged pneumoperitoneum—all of which may have reduced renal reserve and heightened susceptibility to toxic or ischemic insults. Patients with known chronic kidney disease, diabetes, hypertension, or vascular compromise are likely at even greater risk. Until robust safety data exist, intraureteral ICG should be avoided in patients with pre-existing renal impairment or other risk factors for AKI. In lower-risk patients, its use should be carefully weighed against the availability of alternative ureteral visualisation strategies, such as preoperative stenting, lighted catheters, or intraoperative ultrasound [30].

Third, our experience emphasises the importance of perioperative renal monitoring when intraureteral ICG is used. Baseline renal function tests, strict monitoring of urine output, and early postoperative serum creatinine assessment should be standard. In our case, prompt recognition of anuria and early nephrology involvement allowed initiation of hemodialysis before overt uremic complications occurred. This highlights the value of proactive surveillance, which may mitigate morbidity if dye-related nephrotoxicity occurs.

Fourth, there is a strong imperative for systematic reporting of adverse events. To date, the literature has emphasised the benefits of ICG fluorescence without documenting nephrotoxic complications [10,11,31]. It is possible that rare but serious events, such as the one we report, have occurred but remain unpublished due to underreporting or attribution to multifactorial perioperative causes. As the use of intraureteral ICG expands, a registry or collaborative reporting system could provide the necessary denominator to define risk accurately. Even sporadic case reports, such as ours, are valuable in signalling potential hazards and stimulating further study.

Finally, the broader implication is that innovation in fluorescence-guided surgery must be balanced by rigorous safety evaluation. The enthusiasm for enhanced visualisation tools should not outpace evidence regarding toxicological effects. The pharmacology of ICG has been thoroughly studied in systemic use, but assumptions of safety cannot be automatically extended to novel delivery routes. For example, drugs considered safe intravenously may be toxic intrathecally or intraperitoneally. Similarly, intraureteral ICG may expose renal tissues to concentrations never encountered with intravenous dosing. Bridging this knowledge gap requires translational research, including the use of animal models to assess renal histopathology after dye instillation and pharmacokinetic studies of urinary tract absorption.

In practical terms, we propose the following **recommendations for clinicians** considering intraureteral ICG use:

1. **Use the lowest effective dose and concentration** until formal dose–response data are available.
2. **Avoid use in patients with pre-existing renal disease or significant risk factors for AKI**, including chronic hypotension, diabetes, and advanced age.
3. **Implement perioperative renal monitoring protocols**, including urine output assessment and postoperative creatinine measurement.
4. **Engage nephrology early** if oliguria or anuria develops after ICG exposure.
5. **Report all adverse renal events** to institutional safety boards and, where possible, in the medical literature to accelerate collective learning.

This case should not discourage innovation in fluorescence-guided surgery, but rather encourage the careful and evidence-based integration of new applications. If used judiciously and with robust monitoring, ICG may continue to offer significant value in complex gynecologic surgery. However, until its renal safety is clarified, surgeons must remain vigilant and transparent about potential risks.

Although intraureteral ICG administration remains an off-label technique, it can be performed within ethically approved institutional research settings. Centers using this approach should ensure formal board authorization, detailed patient consent, and immediate nephrology support for early detection and management of possible complications

Emerging fluorescence devices, such as the near-infrared ray catheter (NIRC™) and fluorescent ureteral catheter (FUC, IRIS system), represent promising non-pharmacologic alternatives for ureteral visualization. These catheters emit fluorescence intrinsically, providing constant signal intensity independent of urine flow and eliminating dye exposure risk. Clinical series have demonstrated excellent safety and visibility across colorectal, urologic, and gynecologic surgery, including 141 consecutive high-risk colorectal cases with zero ureteral injuries or conversions [32,33]. Similar advantages were reported in laparoscopic ureterectomy [34], ureteroureterostomy after kidney transplantation [35], and pelvic oncologic procedures [36]. Future comparative studies should evaluate ICG-based versus device-based fluorescence to define the optimal approach in different surgical contexts.

### 3.6. Limitations

As a single case, causal inference remains speculative. We could not measure intraureteral pressure or ICG concentration in renal effluent, leaving the mechanism—chemical versus ischemic—uncertain. Additionally, transient perioperative factors such as anemia or pneumoperitoneum could not be fully quantified.

### 3.7. Future Research

Future studies should include animal models to quantify retrograde diffusion of ICG into renal tissue, pharmacokinetic profiling of urinary tract absorption, and multicenter registries tracking postoperative renal function in patients receiving intraureteral ICG.

## 4. Conclusions

We report the first case of acute kidney injury requiring dialysis following intraureteral ICG instillation during bilateral endometrioma excision. Although renal function recovered fully, the event highlights a potential safety concern associated with a technique that is increasingly adopted in minimally invasive surgery. Until systematic safety data are available, intraureteral ICG should be used with caution, particularly in patients at risk of AKI. Surgeons should weigh benefits against possible renal toxicity and ensure close postoperative monitoring.

## Figures and Tables

**Table 1:** Clinical Course and Laboratory Trends.

Day	Event	Key Findings
0	Surgery	Bilateral endometrioma excision; intraureteral ICG 5 mg total
1	Early postop	Anuria; creatinine 2.35 mg/dL; CRP 35.5 mg/L
2	Workup	CT: bilateral renal dysfunction, no obstruction; dialysis initiated
5	Recovery	Urine output improving; creatinine 2.0 mg/dL
7	Oliguria resolving	Urine 1200 mL/day; creatinine 1.35 mg/dL
8	Normalization	Creatinine 0.92 mg/dL; dialysis discontinued
9	Discharge	Stable renal and hematologic parameters

## Data Availability

The original contributions presented in this study are included in the article. Further inquiries can be directed to the corresponding author.

## References

[1] Giudice L.C. (2010). Clinical practice Endometriosis. N. Engl. J. Med..

[2] Dunselman G.A.J., Vermeulen N., Becker C., Calhaz-Jorge C., D’Hooghe T., De Bie B., Heikinheimo O., Horne A.W., Kiesel L., Nap A. (2014). ESHRE guideline: Management of women with endometriosis. Hum. Reprod..

[3] Johnson N.P., Hummelshoj L. (2013). World Endometriosis Society Montpellier Consortium. Consensus on current management of endometriosis. Hum. Reprod..

[4] Vakili B., Chesson R.R., Kyle B.L., Shobeiri S.A., Echols K.T., Gist R., Zheng Y.T., Nolan T.E. (2005). The incidence of urinary tract injury during hysterectomy: A prospective analysis based on universal cystoscopy. Am. J. Obstet. Gynecol..

[5] Park J.H., Park J.W., Song K., Jo M.K. (2012). Ureteral injury in gynecologic surgery: A 5-year review in a community hospital. Korean J. Urol..

[6] Liapis A., Bakas P., Creatsas G. (2001). Ureteral injuries during gynecological surgery. Int. Urogynecol. J. Pelvic. Floor. Dysfunct..

[7] Hope-Ross M., Yannuzzi L.A., Gragoudas E.S., Guyer D.R., Slakter J.S., Sorenson J.A., Krupsky S., Orlock D.A., Puliafito C.A. (1994). Adverse reactions due to indocyanine green. Ophthalmology.

[8] Fox I.J., Wood E.H. (1960). Indocyanine green: Physical and physiologic properties. Proc. Mayo Clin..

[9] Boni L., David G., Mangano A., Dionigi G., Rausei S., Spampatti S., Cassinotti E., Fingerhut A. (2015). Clinical applications of indocyanine green (ICG) enhanced fluorescence in laparoscopic surgery. Surg. Endosc..

[10] Alander J.T., Kaartinen I., Laakso A., Pätilä T., Spillmann T., Tuchin V.V., Venermo M., Välisuo P. (2012). A review of indocyanine green fluorescent imaging in surgery. Int. J. Biomed. Imaging.

[11] Verbeek F.P., van der Vorst J.R., Schaafsma B.E., Swijnenburg R.J., Gaarenstroom K.N., Elzevier H.W., van de Velde C.J., Frangioni J.V., Vahrmeijer A.L. (2013). Intraoperative near infrared fluorescence guided identification of the ureters using low dose methylene blue. J. Urol..

[12] Reinhart M.B., Huntington C.R., Blair L.J., Heniford B.T., Augenstein V.A. (2016). Indocyanine green: Historical context, current applications, and future considerations. Surg. Innov..

[13] Manoucheri E., Cohen S.L., Sandberg E.M., Kibel A.S., Einarsson J. (2012). Ureteral injury in laparoscopic gynecologic surgery. Rev. Obstet. Gynecol..

[14] Siddighi S., Yune J.J., Hardesty J. (2014). Indocyanine green for intraoperative localization of ureter. Am. J. Obstet. Gynecol..

[15] Barnes T.G., Hompes R., Birks J., Mortensen N.J., Jones O., Lindsey I., Guy R., George B., Cunningham C., Yeung T.M. (2018). Methylene blue fluorescence of the ureter during colorectal surgery. Surg. Endosc..

[16] Soriano C.R., Cheng R.R., Corman J.M., Moonka R., Simianu V.V., Kaplan J.A. (2021). Feasibility of injected indocyanine green for ureteral identification during robotic left-sided colorectal resections. Am. J. Surg..

[17] van den Bos J., Al-Taher M., Bouvy N.D., Stassen L.P.S. (2019). Near-infrared fluorescence laparoscopy of the ureter with three preclinical dyes in a pig model. Surg. Endosc..

[18] Keller D.S., Ishizawa T., Cohen R., Chand M. (2017). Indocyanine green fluorescence imaging in colorectal surgery: Overview, applications, and future directions. Lancet Gastroenterol. Hepatol..

[19] Kellum J.A., Lameire N. (2013). KDIGO AKI Guideline Work Group. Diagnosis, evaluation, and management of acute kidney injury. Kidney Int..

[20] Hoste E.A.J., Kellum J.A., Selby N.M., Zarbock A., Palevsky P.M., Bagshaw S.M., Goldstein S.L., Cerdá J., Chawla L.S. (2018). Global epidemiology and outcomes of acute kidney injury. Nat. Rev. Nephrol..

[21] Alford R., Simpson H.M., Duberman J., Hill G.C., Ogawa M., Regino C., Kobayashi H., Choyke P.L. (2009). Toxicity of Organic Fluorophores Used in Molecular Imaging: Literature Review. Mol. Imaging.

[22] Bilgic T., Narter F. (2020). Effects of pneumoperitoneum with carbon dioxide on renal and hepatic functions in rats. Wideochir Inne Tech Maloinwazyjne.

[23] Demyttenaere S., Feldman L.S., Fried G.M. (2007). Effect of pneumoperitoneum on renal perfusion and function: A systematic review. Surg. Endosc..

[24] McDonald J.S., McDonald R.J., Comin J., Williamson E.E., Katzberg R.W., Murad M.H., Kallmes D.F. (2013). Frequency of acute kidney injury following intravenous contrast medium administration: A systematic review and meta-analysis. Radiology.

[25] Pickkers P., Darmon M., Hoste E., Joannidis M., Legrand M., Ostermann M., Prowle J.R., Schneider A., Schetz M. (2021). Acute kidney injury in the critically ill: An updated review on pathophysiology and management. Intensive Care Med..

[26] Balwani M.R., Bawankule C.P., Ramteke V., Tolani P., Vakil S., Yadav R. (2017). Methylene Blue Induced Methemoglobinemia with Acute Kidney Injury in a Glucose-6-Phosphate Dehydrogenase-deficient Patient. Indian J. Nephrol..

[27] Ristea M.E., Zarnescu O. (2023). Indigo Carmine: Between Necessity and Concern. J. Xenobiot..

[28] Kruiswijk M.W., Nguyen D.H.L., Tange F.P., Koning S., van den Hoven P., Peul R.C., Rotmans J.I., Huurman V.A.L., Alwayn I.P.J., Hamming J.F. (2025). The safety of indocyanine green in patients with advanced chronic kidney disease or kidney transplantation: A scoping review. Ann. Med. Surg..

[29] Jiao Y., Liu Y., Jin M. (2024). Exploring the dark side of diagnostic dyes with a focus on Indocyanine green’s adverse reactions. Sci Rep..

[30] Jayasinghe J.D., Butnari V., Hu K.W., Thaha M.A. (2025). Intraoperative techniques in realtime ureteric navigation. A brief narrative review and a video vignette. Front. Surg..

[31] Tanaka Y., Asada H., Kuji N., Yoshimura Y. (2008). Ureteral catheter placement for prevention of ureteral injury during laparoscopic hysterectomy. J. Obstet. Gynaecol. Res..

[32] Plante M., Touhami O., Trinh X.B., Renaud M.C., Sebastianelli A., Grondin K., Gregoire J. (2015). Sentinel node mapping with indocyanine green and endoscopic near-infrared fluorescence imaging in endometrial cancer. A pilot study and review of the literature. Gynecol. Oncol..

[33] Ryu S., Imaizumi Y., Nakashima S., Kawakubo H., Kawai H., Kobayashi T., Ito R., Nakabayashi Y. (2024). Utility and challenges of ureteral visualization using a fluorescent ureteral catheter in high-risk surgeries for colorectal cancer. Surg. Endosc..

[34] Honda T., Matsuoka Y., Osaki Y., Tohi Y., Naito H., Kato T., Okazoe H., Taoka R., Ueda N., Sugimoto M. (2024). Usefulness of fluorescent ureteral catheter during laparoscopic residual ureterectomy. IJU Case Rep..

[35] Takai S., Nishida H., Fukuhara H., Kurokawa M., Tsuchiya N. (2024). Ureteroureterostomy with near-infrared ray catheter in a kidney transplant. Cureus.

[36] Natsume T., Kobayashi-Kato M., Yoshida H., Tanase Y., Uno M., Ishikawa M. (2025). Laparoscopic resection of central ovarian cancer recurrence in the pelvis utilizing a fluorescent ureteral near-infrared ray catheter (NIRC): A case report. Cureus.

